# Towards Genetic Dissection of Skeletal Class III Malocclusion: A Review of Genetic Variations Underlying the Phenotype in Humans and Future Directions

**DOI:** 10.3390/jcm12093212

**Published:** 2023-04-29

**Authors:** Osayd Zohud, Iqbal M. Lone, Kareem Midlej, Awadi Obaida, Samir Masarwa, Agnes Schröder, Erika C. Küchler, Aysar Nashef, Firas Kassem, Vadim Reiser, Gavriel Chaushu, Richard Mott, Sebastian Krohn, Christian Kirschneck, Peter Proff, Nezar Watted, Fuad A. Iraqi

**Affiliations:** 1 Department of Clinical Microbiology and Immunology, Sackler Faculty of Medicine, Tel-Aviv University, Tel-Aviv 6997801, Israelkareemmidlej@mail.tau.ac.il (K.M.); 2 Center for Dentistry Research and Aesthetics, Jatt 4491800, Israel; 3 Department of Orthodontics, University Hospital of Regensburg, University of Regensburg, 93047 Regensburg, Germany; 4 Institute for Clinical Microbiology and Hygiene, 93053 Regensburg, Germany; 5 Department of Oral and Maxillofacial Surgery, Baruch Padeh Medical Center, Poriya, Tabaria 1520800, Israel; 6 Department of Otorhinolaryngology, Head and Neck Surgery, Meir Medical Center, Kfar Saba 4428164, Israel; 7 Sackler Faculty of Medicine, Tel-Aviv University, Tel-Aviv 6997801, Israel; 8 Department of Oral & Maxillofacial Surgery, Rabin Medical Center, Beilinson Campus, Petah Tikva 4941492, Israel; 9 School of Dental Medicine, Tel-Aviv University, Tel-Aviv 69978, Israel; 10 Department of Genetics, University College of London, London SE1 7EH, UK; 11 Department of Orthodontics, Faculty of Dentistry, Arab America University, Jenin 34567, Palestine; 12 Gathering for Prosperity Initiative, Jatt 4491800, Israel

**Keywords:** quantitative trait loci (QTL) mapping, genome-wide association study (GWAS), epigenetics-wide association study (EWAS), RNAseq analysis, expression quantitative trait loci (eQTL), micro and small RNA analysis

## Abstract

Introduction: Skeletal abnormalities and malocclusions have varied features that impact populations globally, impairing aesthetics and lowering life quality. The prevalence of the Skeletal Class III disease is the lowest among all angle malocclusions, with varied prevalence across nations. Environmental, genetic, and societal factors play a role in its numerous etiologies. In this study, we conducted a thorough search across the published data relating to quantitative trait loci (QTL) and the genes associated with Class III progression in humans, discussed these findings and their limitations, and proposed future directions and strategies for studying this phenotype. Methods: An inclusive search of published papers in the PubMed and Google Scholar search engines using the following terms: 1. Human skeletal Class III; 2. Genetics of Human skeletal Class III; 3. QTL mapping and gene associated with human skeletal Class III; 4. enriched skeletal Class-III-malocclusion-associated pathways. Results: Our search has found 53 genes linked with skeletal Class III malocclusion reported in humans, genes associated with epigenetics and phenomena, and the top 20 enriched pathways associated with skeletal Class III malocclusion. Conclusions: The human investigations yielded some contentious conclusions. We conducted a genome-wide association study (GWAS), an epigenetics-wide association study (EWAS), RNA-seq analysis, integrating GWAS and expression quantitative trait loci (eQTL), micro- and small-RNA, and long non-coding RNA analysis in tissues connected to skeletal Class III malocclusion phenotype in tissues connected with the skeletal phenotype. Finally, we invite regional, national, and international orthodontists and surgeons to join this effort by contributing human samples with skeletal Class III malocclusion following the accepted Helsinki ethical protocol to challenge these phenomena jointly.

## 1. Introduction

Around the world, populations are affected by a variety of skeletal conditions, including malocclusions, leading to reduced language ability, aesthetic quality, and lower quality of life [1]. Malocclusion is one of the top three oral health issues, according to the World Health Organization (WHO), behind periodontitis and caries [2]. According to estimates, the incidence varies from 39% to 93% in kids and teens [3]. The spectrum and clinical heterogeneity were highest in Asians and low among Caucasians [4], while in the Middle East, Class III malocclusion is more frequent than in Caucasians but less common than in Far Eastern Asians [4,5,6]. The least frequent Class of Angle malocclusion is Class III, with a mean frequency of 7.2%. Reports have shown that the countries with the lowest prevalence index were Italy (1.6%), Nigeria (1.6%), and Jordan (1.4%). The prevalence of Class I malocclusion ranges from 50 to 55%, as opposed to 15% and 1%, respectively, for Class II and Class III malocclusion, according to recent reports. In the US, 30% of people have a normal occlusion [7]. Normal occlusion is prevalent in Denmark at 14%, but Class I, Class II, and Class III malocclusions have been reported to be prevalent at 58%, 24%, and 4%, correspondingly [8]. Similar to any other condition, skeletal malocclusions and/or deformities have a complex etiology, which are often related to sociological, genetic, and environmental factors [9]. Both molar and canine associations of malocclusion have been documented in earlier research [10]. 

According to research, malocclusion is a complicated disorder produced by the combination of several variables, such as heredity, environment, and individual actions [11]. Recent studies have highlighted evidence of the genetic role in the development of malocclusion [12]. Studies have shown that malocclusion has a hereditary component, and genetic factors can contribute to the development of distinct forms of malocclusion [13]. Although the precise mechanisms underlying these genetic impacts remain unclear, detecting phenotype–genotype correlations may help identify specific genetic abnormalities linked to certain types of malocclusions [11]. This review aims to provide an overview of the current state of knowledge regarding the genetic influence on malocclusion, focusing on the potential role of genetics in the formation of malocclusions, indicating that various genes and genetic pathways are associated with distinct forms of malocclusion. By understanding these relationships better, we aim to develop more effective treatment options that target the underlying genetic causes of malocclusion rather than merely addressing the symptoms. The study of genetics in malocclusion is still in its early stages, and much more research is needed to properly understand the dynamic interplay of genetics, environment, and individual behavior in the development of this disorder. However, a growing body of research shows that heredity plays an essential role in malocclusion and that by better understanding the genetic impacts on this problem, we may assist in enhancing diagnosis, treatment, and preventive efforts.

### Objectives

In this study, skeletal variations and potential craniofacial genes are evaluated in patients with skeletal malocclusions. To identify phenotype–genotype relationships that have clinical significance, the study aims to explore the current understanding of the genetic factors involved in the development of this condition. The review describes the existing research on the potential craniofacial genes and loci related to the craniofacial skeleton, as well as explores the genetic pathways involved in anteroposterior and vertical skeletal variations in these patients. Furthermore, the review aims to discuss the implications of these findings for the diagnosis and treatment of Class III malocclusion. Specifically, the review seeks to identify potential therapeutic targets based on the genetic factors associated with this condition, which could lead to more effective treatments for affected individuals. It is suggested that future research should look at the variations in soft tissues as well as skeletal structures in malocclusion patients to determine the hereditary causes of these variances. We will gain a better understanding of the molecular controls over postnatal facial growth and inform the development of personalized treatment plans based on an individual’s genetic profile, which will help us treat individuals with malocclusion more effectively.

Altogether, this review will attempt to offer a complete summary of the present state of knowledge on the link between genotype and phenotype in Class III malocclusion, as well as to propose potential future study and treatment interventions.

## 2. Methods

The PRISMA guidelines for systematic meta-analyses and reviews, the 2009 checklist, and the GRADE guidelines, were all adhered to in this study. From early 1990 to January 2022, we looked for reports that described the genetic or epigenetic aspects of skeletal malformations and malocclusions. Using the following criteria for inclusion, we found studies that were appropriate: (1) original research or meta-analysis; (2) English language writing; (3) Human skeletal Class III; (4) Genetics of Human skeletal Class III; (5) QTL mapping and gene association with human skeletal Class III; and (6) enriched skeletal Class-III-malocclusion-associated pathways. The following studies were excluded from consideration: (1) mammal, histopathologic, in vitro, or computational studies; (2) reviews; (3) transcriptomic or expression studies without epigenetic/genotyping analysis; (4) reports focusing on other conditions and malocclusions were simply discussed; and (5) reports for which we did not have access to the full text or that were written in another language. 

In the months of May and June 2022, a study was carried out using PubMed (National Library of Medicine, 8600 Rockville Pike, Bethesda, MD, USA 20894) and the Google Scholar search engines (https://scholar.google.com) using the following terms: Human skeletal Class III; Genetics of Human skeletal Class III; QTL mapping and genes associated with human skeletal Class III; enriched skeletal Class III-malocclusion-associated pathways; and genome-wide association study. 

Three authors separately analyzed the database search results, appraised the titles and abstracts, and thought about thoroughly reviewing the paper. Any differences were settled by consensus during either the title/abstract or full manuscript review phases. This systematic review included formal evaluations of each qualifying study. The authors appraised the included studies’ quality assessment and bias risk separately.

## 3. Results

### 3.1. Clinical Outcomes of the Phenotype and Their Direct Impact on the Treatment

**Figure 1:**
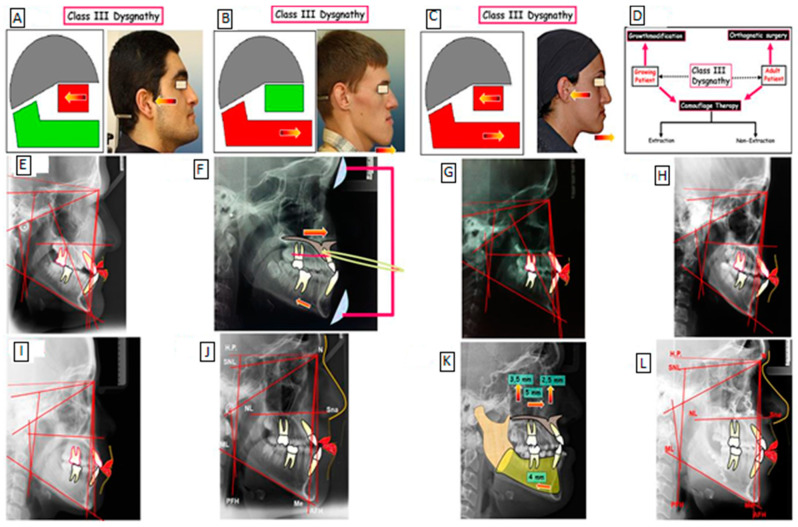
Class III skeletal malocclusion phenotype is characterized by maxillary (midface) retrusion (**A**), mandibular protrusion (mandibular prognathism; (**B**), a combination of maxillary retrusion and mandibular protrusion (mandibular prognathism; (**C**). Treatment strategies for Class III dysgnathy (**D**). Treatment of Class III dysgnathy in the growing age through growth influencing methods (**E**–**G**): before treatment (**E**); use of devices to influence growth (**F**); after treatment (**G**). Camouflage therapy of Class III dysgnathy by extraction of permanent teeth (**H**,**I**): before treatment (**H**); after treatment (**I**). Combination therapy (orthodontics and surgery) of Class III dysgnathy (**J**–**L**): before treatment (**J**); Simulation of surgical maxillary-mandibular movement (**K**); after treatment (**L**).

The skeletal Class III malocclusion phenotype is diverse and typically defined by maxillary (midface) retrusion (Figure 1A), mandibular protrusion (mandibular prognathism), or both (Figure 1B,C). These features may be present alone or as a component of a syndrome [14]. Individuals affected by skeletal Class III malocclusions (maxillary deficiency and mandibular prognathism) caused by an underlying skeletal anomaly have additional issues that adversely affect physical, social, and psychological well-being. Early detection of the phenotypic is possible, and it typically becomes more obvious as an adult grows, with a stronger prevalence in females [15].

Skeletal Class II and Class III deformities considerably impair patients’ capability to chew food effectively. Additionally, Class III skeletal malocclusion probands have a high level of abruptness, irregular chewing movements, and a compromised bolus swallowing ability [16]. Class II and III skeletal deformities can also impair other digestive tract parts’ roles [17]. In addition, Class II and III skeletal malocclusions are associated with a variety of conditions affecting oral health and gastroesophageal reflux disease [17] and are known to be visually unattractive features from a cosmetic perspective [18], they may also have an impact on a patient’s psychological health and diminish their sense of self-worth. On the other hand, surgical correction can greatly boost their self-esteem and reduce their concerns about their appearance [18]. Class II and III skeletal deformities drastically impact people’s life quality adversely [19]. Interestingly, one or more of these malocclusions affects around 25% of the general population [20].

Skeletal diagnosis is a critical process in determining the most suitable treatment for a patient based on their growth potential [21]. The different phenotypic features of Class III malocclusion have different effects on treatment planning, such as the presence of anterior crossbite, alveolar bone thickness, and Class III malocclusion. For instance, patients with a Class III malocclusion require orthognathic surgery, which retracts the incisors, but distalization and interproximal reduction therapy can be used to camouflage the malocclusion, and multiloop edgewise archwire (MEAW) can be used for mild to moderate anterior crowding [21]. Treatment decisions, such as whether to extract teeth or use skeletal anchorage, are crucial in determining the most effective treatment for skeletal patients. Biomechanics is essential in leveling the dental arch, and the strength of specific muscles can be enhanced or diminished to alleviate long-term relapse caused by abnormal muscular activity [21]. The formation of a gingival cleft is a common consequence of orthodontic space closure, and the lower arch’s distalization potential is often limited due to the difficulty of additional distalization after the distolingual root of the second molar contacts the mylohyoid ridge, increasing the chance of relapse. To avoid undesirable tooth movements, stiff archwires and minimal force can be used. Overall, understanding the different phenotypic features is essential in determining the most effective treatment for patients [21]. Moreover, a better understanding of the genetic etiology of Class III malocclusion can inform treatment decisions, such as selecting the appropriate type of orthodontic appliance or surgery and the duration and intensity of treatment. Overall, a comprehensive understanding of both genetic and phenotypic factors in Class III malocclusion can have significant implications for treatment outcomes and planning, allowing for more personalized and effective treatment approaches.

### 3.2. Mechanical and Surgical Treatments of Skeletal Class III Malocclusion 

Currently, to our knowledge, there are three procedures for correcting a skeletal Class III malocclusion, including I. growth modification, II. orthodontic camouflage therapy, and III. surgical orthodontics, these treatment strategies for Class III dysgnathy are shown in Figure 1. The treatment of Class III dysgnathy in terms of growing age through the use of growth-influencing methods using devices to affect change has been used (Camouflage therapy of a Class III dysgnathy by extracting the permanent teeth and combination therapy (orthodontics surgery).

Skeletal Class III jaw discrepancies in children can be effectively fixed through growth adjustment with dentofacial orthopedic equipment [22]. In terms of case selection therapy, the following characteristics will help in the effective implementation of orthodontic camouflage treatment: (1) regular or narrow facial patterns, (2) a modest anteroposterior jaw discrepancy, (3) a displacement of no more than 4–6 mm, (4) normal soft tissue characteristics, and (5) no transverse skeletal issues. Others used a receiver–operating characteristic analysis of cephalometric variables to classify Class III skeletal malocclusions requiring orthognathic surgery. Four of these six dimensions should be indicated for surgical treatment: (1) overjet, 4.73 mm; (2) Wit’s rating, 11.18 mm; (3) L1-MP angle 80.8; (4) Mx/Mn ratio 65.9%; (5) overbite, 0.18 mm; and (6) gonial angle, 120.8.6. In this patient, only two of these six measurements (L1-MP angle = 79; gonial angle = 122) met the surgical indication.

The above devices can be massive, unpleasant, and only applicable to adolescent patients. Anterior crossbite has been successfully treated in adults using braces with Class III rubber bands and plastic adhesive bites for disocclusion. The supra-eruptive lower anterior teeth are likewise pushed into with anterior resin bites. Prior to the treatment, the upper teeth were insufficient. The crowding of the upper teeth was removed, and an attractive smile curve was also accomplished by enlarging the upper front incisors. When the patient smiles, the upper lip is supported by the upper anterior teeth gum line. Tooth exposure exceeds the suggested 0 to 2 mm of the lip coverage of the frontal teeth for posed smiles for the female Asian average [23]. The following factors are responsible for the few changes to the lower incisors: (1) light force and brief spacing of Class III elastics (3/16 2 oz) were used, as were small NiTi wires (0.013/0.014 inch) for the initial work, in order to avoid the undesirable side effect of the excessive withdrawal of the lower front teeth; (2) when closing the lower interdental space, a gradual increase in the dimensions of the orthodontic wires with a suitable torque on the buccal crown of the lower front teeth; and (3) a preloaded NiTi wire combination for controlling the torque on the mandibular anterior teeth (0.017 × 0.025 NiTi with 20 degrees of lingual root torque). The mandibular second premolars, followed by the mandibular and maxillary lateral incisors, are the most common teeth not present at birth [24]. Dental agenesis is hypothesized to be affected by genetic and environmental influences, or a combination of both, which interfere with tooth development. Several studies have found that patients with dental agenesis typically have smaller teeth than patients without dental agenesis [25]. There are two available treatments to close the missing tooth gap: Firstly, close the gaps and let the permanent first molar drift mesially before finishing the gap closure orthodontically. Secondly, maintain or regain the spaces for the prosthesis [26].

At the completion of skeletal Class III molar occlusion, the patient should be checked for the presence of antagonist teeth that can occlude the maxillary second molars, such as mandibular third molars. To achieve a good Class III molar relationship, some adjustments might be needed, such as moving the mandibular first molars more lingually than usual, not having the lower first molar offset, having the upper premolars and molars offset more, not having the maxillary molars toe in, applying lingual crown torque to the mandibular molars, and reducing the palatal crown torque in the maxillary premolars. For better intercuspation, some contouring or occlusal adjustment was needed, such as reducing the palatal cusps of the upper premolars and molars or enhancing the buccal cusps of the lower molars with restoration [27]. 

Class III malocclusion can be a challenging task in orthodontic therapy as there is a need to differentiate between dental and skeletal Class III malocclusion. The criteria for determining whether a patient has a Class III dental or skeletal malocclusion and the criteria for achieving successful camouflage treatment outcomes were examined. The Class III malocclusion patient can be cured better with proper examination and camouflage treatment.

### 3.3. Current Constraints of Genetic Analysis

Here we discuss the factors that contribute to condylar growth under mechanical stress and the genes that may play a role in the development of Class III malocclusion. Any suggested gene involved in this biological pathway could be a candidate gene related to Class III malocclusion. Recent reports related to genetic studies using linkage analysis and association studies have identified genes that confer susceptibility to Class III malocclusion [28,29]. Knowing that genetic factors may play a role in the etiology of skeletal Class III malocclusion, it is important to identify gene variants that predispose individuals to the condition in order to predict their likelihood of developing it and aid in early prevention or treatment.

These earlier genetic studies have several drawbacks, including small sample sizes, the exclusion of environmental factors, a lack of a systematic estimate of the genetic variants linked to the disease, and, perhaps more importantly, limitations in defining the phenotypes that do not reflect the complexity of Class III phenotypes. [11]. Class III malocclusion remains difficult for dentists to diagnose and treat due to the limited understanding of the underlying etiologies of this condition. The first step in identifying the genetic component of Class III malocclusion is to distinguish between the phenotypes associated with various genotype expressions.

Furthermore, several genes and loci have been linked to mandibular prognathism through various genetic linkage analyses and genome-wide association studies. The search for new candidate genes is aided by the ambiguity surrounding the genes that influence the risk of mandibular prognathism in the general population. Due to variations in genetic background, one population’s genetic association might not translate to another population. Therefore, replication studies need to be performed in order to ascertain the genotype and allele distribution in various human populations, providing data that might be used as a basis for future research. Identifying the genetic factors and understanding their mechanisms with Class III malocclusion development would aid in the diagnosis, prediction, and treatment of skeletal variations. Single-nucleotide polymorphisms (SNPs) and the haplotypes defined by shared SNPs can be genotyped to determine normal and variable craniofacial phenotypes [30]. Studies have shown that some SNPs (P561T, C422F, and I526L) occurring in the growth hormone receptor (GHR) gene are associated with mandibular ramus height in Japanese, Korean, and Chinese populations, and the P561T polymorphism has an inhibitory effect on mandibular growth in young children [28]. Multiple SNPs have been found to be involved in mandibular prognathism [31]. An SNP of the noggin gene (rs1348322) was present in four families with mandibular micrognathia [32].

### 3.4. Genome-Wide Association Studies (GWAS)

Five genome-wide nonparametric linkage studies and one genome-wide association study (GWAS) for detecting the loci related to mandibular prognathism and the skeletal Class III phenotype recently yielded contradictory results. From the results of the linkage analysis by Yamaguchi et al. [28], mandibular prognathism has been linked to chromosomes 1p36, 6q25, and 19p13.2 in Japanese and Korean families. Association studies surrounding 1p36 revealed that matrilin-1 (cartilage matrix protein, [31]) and EPB41 (erythrocyte membrane protein band 4.1; [12]) were potential phenotypic genes. Another linkage analysis of Brazilian families, on the other hand, discovered that 1p36, 6q25, and 19p13.2 were not related to this trait. [33]. In addition, the results of the linkage analysis of Colombian Hispanic families by Frazier-Bowers et al. [29] proposed that five susceptibility loci, 1p22.1, 3q26.2, 11q22, 12q13.13, and 12q23, are linked to the phenotype. As an alternate technique for linkage analysis, GWAS has been proposed [34,35]. There have been two studies GWASs in relation to mandibular prognathism, with Yamaguchi et al. [28] and Frazier-Bowers et al. [29] revealing that the vulnerability loci for mandibular prognathism were on the first chromosome. In a Brazilian population, the SNPs rs708111 (*WNT3A*), GLI2 rs3738880, and FGF3 rs1893047, were associated with skeletal Class III malocclusion, and SNP rs3934908 (*SMAD6*) was associated with prognathism [36].

A GWAS of mandibular prognathism utilizing microsatellites was undertaken initially, and six loci (1p22.3, 1q32.2, 3q23, 6q23.2, 7q11.22, and 15q22.22) were found as susceptibility areas related to mandibular prognathism. The candidate genes include SSX2IP, PLXNA2, RASA2, TCF21, CALN1, and RORA. A prior linkage investigation validated the 1p22.3 locus, and five additional loci (1q32.2, 3q23, 6q23.2, 7q11.22, and 15q22.22) were discovered.

To identify the susceptibility sites for mandibular prognathism, the first GWAS using microsatellites was conducted on Japanese patients, including 240 subjects with mandibular prognathism and 360 healthy subjects [37]. At chromosomes 1p36, 6q25, and 19p13.2 in humans, genome-wide linkage analysis revealed evidence of a linkage to mandibular prognathism [28]. The presence of the P56IT variant in the growth hormone receptor gene has been shown to have an impact on mandibular morphology in Chinese and Japanese populations, particularly in relation to mandibular height. Research on a Hispanic cohort indicated that Class III (mostly caused by maxillary regurgitation) is transmitted in an autosomal dominant pattern and is linked to five genetic markers: 1p22.1, 3q26.2, 11q22, 12q13.13, and 12q23 [29].

Exome sequencing research in families from Estonia [38], China [39], and Italy [40] has identified potential genes that cause mandibular prognathism. However, inconsistent genes within exome-sequencing for the phenotype have also been shown. These discrepancies appear to be caused by separate sub-phenotypes, such as maxillary hypoplasia, mandibular hyperplasia, or a mix of the two in the case of mandibular prognathism [29].

To our knowledge, at the time of writing this study, there are 53 genes linked with skeletal Class III malocclusion, as presented in Table 1. As expected, with the aid of the Reactome database, it was possible to determine that the majority of these genes were enriched in the signaling pathways involved in the control and growth of bone and cartilage. Remarkably, several muscle-related genes and signaling pathways have also been identified, which is consistent with the functional matrix theory since the muscles surrounding the jaw may also contribute to the development of both types of malocclusions.

#### 3.4.1. Epigenetic and Skeletal Class III Malocclusion

Epigenetic regulation regulates gene expression through methods such as DNA methylation, RNA interference, and histone changes [41]. DNA is bundled with histone proteins in eukaryotic cells to create nucleosomes, which are then condensed into chromatin fibers [42]. Acetylation of histones in masseter muscle genes can boost the production of type IIX MHC, which is involved in fast-twitch muscle fibers [43,44]. On the other hand, histone deacetylation can cause chromatin closure, lowering the expression of type I MHC, which regulates slow-twitch muscle fibers [45]. Patients with Class III malocclusion have higher levels of HDAC4 and KAT6B gene expression in their masseter muscle, which is responsible for malocclusion and musculoskeletal complexes [44,45]. KAT6B stimulates RUNX2, which encodes the osteogenic transcription factor, and its epigenetic control is critical for gene expression regulation. Epigenetic studies have revealed that increasing KAT6B expression is related to reduced type IIX MHC expression, indicating a function in muscle fiber type regulation [45].

Recent studies have revealed the importance of various genes encoding particular growth factors and signaling molecules in craniofacial development and the genesis of Class III malocclusion [12]. Among these genes, insulin-like growth factor 1 (IGF1) is of particular interest due to its location in the 12q23 junction region and its essential role in skeletal growth and normal bone metabolism through the GH/GHR/IGF1 system [46]. Additional growth factors, including EGF, HGF, NGF, and PDGF, have been reported to activate ERKs during development and, in adult tissues, increase the transcription of other DUSP family members, which may compensate for DUSP6 deficiency in knockout animals. Despite the fact that numerous growth factors can promote dusp6, a transcriptional link between FGF and DUSP6 may exist [47]. The transcriptional activation of dusp6 by FGF is critical in essential developmental processes, especially in the early stages. Its activation can be precisely controlled by particular FGF/FGFR signaling pathways and FGFR-specific response elements in the regulatory domains of the dusp6 gene. FGF/FGFR signaling may potentially control transcription factor access to dusp6 promoter regions via distinct epigenetic processes and chromatin changes, as previously demonstrated in other genes [48].

The modern paradigm of individualized therapies stresses prevention over cure. Precision medicine focuses on each patient’s DNA as well as an epigenomic factor that impacts the expression of the next individual gene [49]. As a result, genomic research brings up new opportunities to utilize scientific discoveries in the clinic [50]. Personal therapies are emphasized in terms of prevention rather than cure. Precision medicine focuses on each patient’s DNA as well as epigenomic factors that regulate gene expression. As a result, genetic research brings up new opportunities for the clinical application of scientific discoveries [51]. Patient replies decide the coordinator’s directions for inhibiting natural growth and/or deciding if imitation, function, orthopedic prayer, or surgery are useful, in particular, 050010-4 instances [12,51,52]. Furthermore, local variables that influence muscle masculinization gene expression are a possible method of re-programing ions before surgical correction and reducing surgical recurrence [45,50].

#### 3.4.2. Differential Expression Levels of RNA Skeletal Class III Malocclusion

The proposed genes include myosin heavy chain (MYH1, MYH2, MYH3, MYH7, MYH8), forkhead box O3 (FOXO3), nuclear factor of activated T-cells 1 (NFATC1), prostaglandin-endoperoxide synthase 2 (PTGS2), lysine acetyltransferase 6B (KAT6B), histone deacetylase 4 (HDAC4), and runt-related transcription factor 2 (RUNX2) are thought to play a role in epigenetic control that determines how mandibular prognathism manifests in terms of its phenotype and progression [53,54]. According to the suggested theory, some environmental stimuli, such as pressure acting on the mandible, alter the epigenetic processes that govern the expression of genes whose transcripts take part in mandibular development and regulation. The amount of epigenetic control in malocclusion patients is unknown. It is challenging to determine whether bone, muscle, or soft tissue matrices are the main factors influencing craniofacial growth at a specific site due to the complexity of tissue–cell interactions [54].

Gedrange et al. [55] used the competitive RT-PCR assay method to examine the transcription levels for type I and type II myosin heavy chain (MyHC) in masseter 10 participants’ muscle samples, which were separated into two groups (with a skeletal pattern of Class II or Class III), before and six months after orthognathic surgery. Type I and type II MyHC mRNA levels decreased by approximately 87% in both groups, with patients in the mesial mandibular position having a slightly higher deficiency. Harzer et al. [56] investigated the same topic by comparing the transcription levels for MyHC type I, IIa, and IId/x (encoded by MYH7, MYH2, and MYH1, respectively) in masticatory muscle samples from 16 patients with prognathism and 14 patients with retrognathism before and six months after surgery. MyHC type I mRNA levels decreased from approximately 46% to 37% in both groups, while MyHC type IIa mRNA levels increased from approximately 29% to 42%; however, there was no discernible difference among pre- and postoperative mRNA levels for MyHC type IId/x. Furthermore, there was no discernible variance in postoperative MyHC mRNA levels between prognathic and retrognathic patients.

Furthermore, Maricic et al. [57] examined the mRNA levels for mechano-growth factor, myostatin, and MyHC types I, IIa, and IIx/d in masseter muscle samples from 14 participants with prognathic mandibles (ANB angle 48) before and six months after surgical correction. Both groups showed a significant postoperative increase in mechano-growth factor mRNA levels. On the other hand, myostatin mRNA levels did not differ significantly between the before- and after-surgery periods. Importantly, in each cohort, after surgery, MyHC type I mRNA levels were lower than before surgery, whereas postoperative MyHC type IIa levels were higher. There was no discernible variance in mRNA levels for MyHC type IId/x before and after surgery. MyHC type IIa had a greater increase in mRNA levels. Oukhai et al. [58] were interested in the changes in the transcription of the embryonic (type 3) and perinatal MyHC developmental types before and after surgery (type 8). They investigated the masseter muscle of 24 subjects, 11 of whom had mandibular prognathism, before and after surgery (ANB angle 38). MYH8 gene expression was two times higher in the prognathic group than in the retrognathic group, although MYH3 gene expression was the same in both groups.

**Table 1:** The list of identified skeletal Class III malocclusion-associated genes.

Frequency of Gene in Search	Gene Symbol	Name of Gene	Reference (s)
4	*MATN1*	*matrilin 1*	[28,31,59,60]
2	*COL2A1*	*collagen type II alpha 1 chain*	[61,62]
	*FGFR2*	*fibroblast growth factor receptor 2*	[63,64]
	*KAT6B*	*lysine acetyltransferase 6B*	[44]
	*MYO1H*	*myosin IH*	[65,66]
	*PLXNA2*	*plexin A2*	[29,67]
	*SSX2IP*	*SSX family member 2 interacting protein*	[37,68]
1	*ADAMTS1*	*ADAM metallopeptidase with thrombospondin type 1 motif 1*	[69]
	*ADAMTSL1*	*ADAMTS like 1*	[70]
	*ALPL*	*alkaline phosphatase, biomineralization associated*	[28]
	*ARHGAP21*	*Rho GTPase activating protein 21*	[40]
	*BEST3*	*bestrophin 3*	[71]
	*C1orf167*	*chromosome 1 open reading frame 167*	[72]
	*CALN1*	*calneuron 1*	[37]
	*COL1A1*	*collagen type I alpha 1 chain*	[63]
	*DUSP6*	*dual specificity phosphatase 6*	[38]
	*EP300*	*E1A binding protein p300*	[73]
	*EPB41*	*erythrocyte membrane protein band 4.1*	[12]
	*ERLEC1*	*endoplasmic reticulum lectin 1*	[74]
	*EVC*	*EvC ciliary complex subunit 1*	[75]
	*EVC2*	*EvC ciliary complex subunit 2*	[75]
	*FGF12*	*fibroblast growth factor 12*	[76]
	*FGF20*	*fibroblast growth factor 20*	[76]
	*FGF23*	*fibroblast growth factor 23*	[77]
	*FGF3*	*fibroblast growth factor 3*	[78]
	*FGFR1*	*fibroblast growth factor receptor 1-A*	[76]
	*FOXO3*	*forkhead box O3*	[79]
	*GHR*	*growth hormone receptor*	[80]
	*GLI2*	*GLI family zinc finger 2*	[81]
	*HDAC4*	*histone deacetylase 4*	[44]
	*HOXC*	*homeobox C cluster*	[29]
	*HSPG2*	*heparan sulfate proteoglycan 2*	[28]
	*IGF1*	*insulin-like growth factor 1*	[29]
	*JAG1*	*jagged canonical Notch ligand 1*	[73]
	*LTBP2*	*latent transforming growth factor beta binding protein 2*	[82]
	*MMP13*	*matrix metallopeptidase 13*	[29]
	*MYH1*	*myosin heavy chain 1*	[79]
	*MYH8*	*myosin heavy chain 8*	[79]
	*NBPF8*	*NBPF member 8*	[72]
	*NBPF9*	*NBPF member 9*	[72]
	*NCOR2*	*nuclear receptor corepressor 2*	[73]
	*NFATC1*	*nuclear factor of activated T cells 1*	[79]
	*NOTCH3*	*notch receptor 3*	[73]
	*NOTCH4*	*notch receptor 4*	[73]
	*NUMB*	*NUMB endocytic adaptor protein*	[73]
	*PSEN2*	*presenilin 2*	[73]
	*RASA2*	*RAS p21 protein activator 2*	[37]
	*RORA*	*RAR related orphan receptor A*	[37]
	*SMAD6*	*SMAD family member 6*	[36]
	*TBX5*	*T-box transcription factor 5*	[63]
	*TCF21*	*transcription factor 21*	[37]
	*TGFB3*	*transforming growth factor beta 3*	[82]
	*WNT3A*	*Wnt family member 3A*	[36]

## 4. Discussion

### 4.1. Gene Identifications and Validations

Class III malocclusion is a complex maxillofacial disorder that has long been recognized as being characterized by a curved shape, revealing mandibular protrusion, maxillary retrusion, or a mix of the two [83], and the malocclusion’s possible anatomical heterogeneity. Lateral cephalometric radiographs reveal cranial, facial bony, and soft tissue structures and are a rich source of phenotypic information. Cephalometric analysis is a low-cost and practical adjunctive study that is primarily used to define phenotypes in and within the Class III population [4,84]. Lately, several studies have used multivariate analysis such as discriminant analysis, principal component analysis (PCA), and cluster analysis to distinguish the phenotypic variations of Class III malocclusion. PCA is a potent technique for obtaining a summary of complicated multivariate data [85]. Instead, cluster analysis adds to the PCA-organized variables to pick homogeneous data, allowing the identification of the underlying phenotype. The subtypes of other diseases have also been identified using this technique [86]. Although earlier research has helped to characterize Class III malocclusion, it is not yet clear whether the phenotypic classifications for this group can be applied to other samples and populations. Future genetic studies will benefit from this research, which will help with clinical diagnoses. In total, 53 genes were discovered through data mining that were considered to be related to skeletal Class III malocclusion (Table 1). Four independent studies listed matrilin 1 gene (MATN1); collagen type II alpha 1 chain (COL2A1), fibroblast growth factor receptor 2 (FGFR2), lysine acetyltransferase 6B (KAT6B), myosin IH (MYO1H), and plexin A2 genes (PL 44 genes associated with skeletal Class III malocclusion, were found in the Reactome database. This is relevant to pathway enrichment. Table 2 lists the top 20 enriched pathways among them.

### Future Directions for Thoroughly Analyzing the Complex Human Class III Malocclusion Genes

In order to comprehend the genetic elements that might play a role in the initiation, advancement, and establishment of skeletal Class III malocclusion, a new Class III malocclusion model and approach should be developed considering the diversity of the human population systems genetic analysis has provided the first comprehensive overview of complex traits’ molecular makeup. It is helpful in the identification of genes, signaling pathways, and networks that underlie common diseases. Data related to cellular, molecular, and clinical features were then analyzed to determine the correlations of various skeletal Class III malocclusion phenotypes.

**Figure 2:**
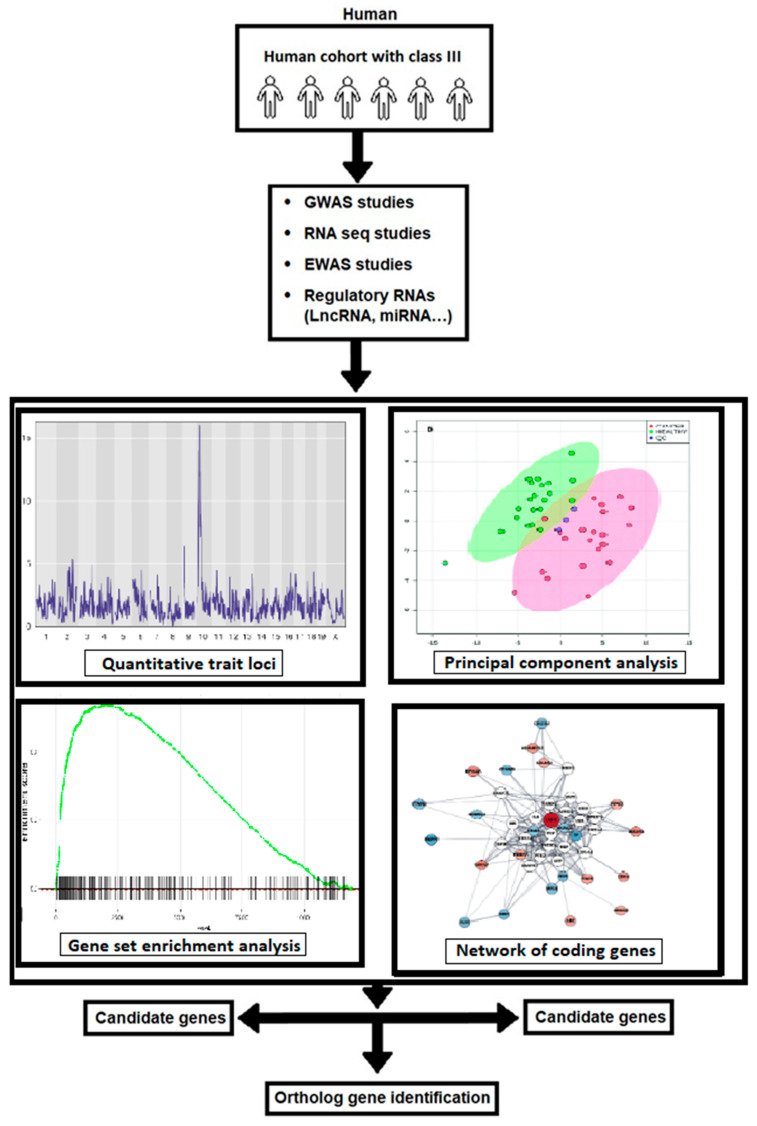
The workflow of the generation of system genetic datasets of cellular, molecular, and clinical trait data combined to analyze various correlations between malocclusion and Class III phenotypes. By integrating SNP genotype data, the regulatory genomic regions are implicated in phenotypic variation in both; in vitro and in vivo monitored traits can be identified using QTL mapping. Combining data with subsequent candidate gene association studies in humans has the potential to identify susceptibility genes associated with the development of Class III malocclusion in man.

**Figure 3:**
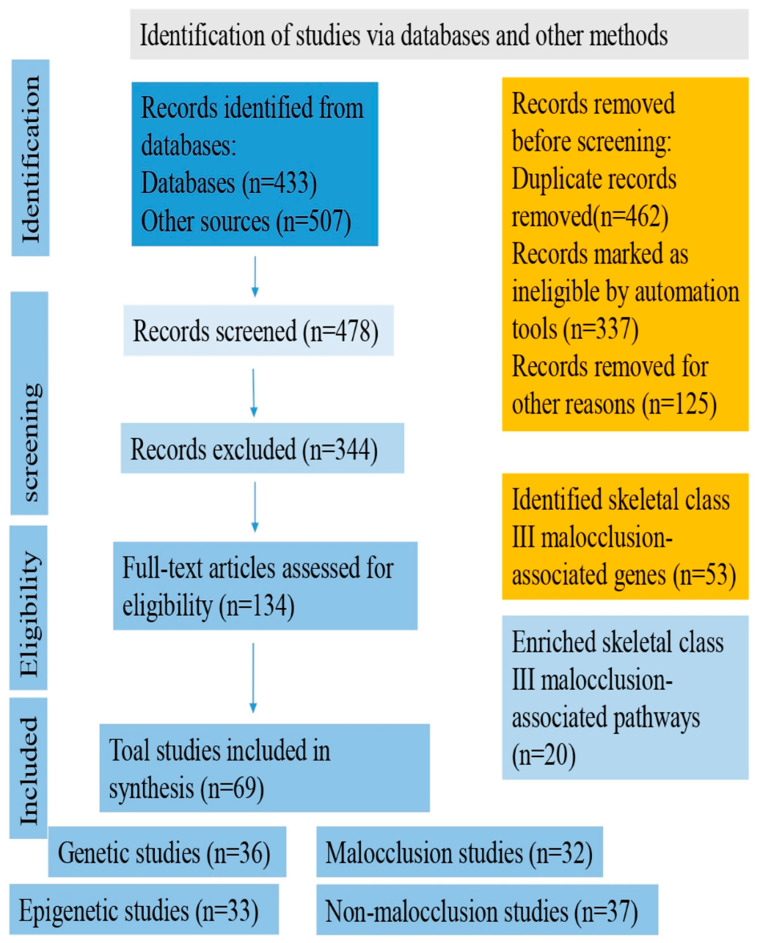
Flow diagram of the selection process of Class III malocclusion in man.

The advancement of high-throughput assessment technology and computational methodologies could lead to a better understanding of how the interaction of multiple genetic changes impacts the beginning and severity of Class III malocclusion. Proven gene–gene interactions and/or gene–environment networks may be used to assess risk in humans for Class III malocclusion avoidance or pharmacological targets. Knowing the functions of the genetic loci systems’ genetics, could most probably have the ability to appreciate the molecular basis and severity of the illness thanks to the genes discovered in the genome-wide association study (GWAS) that increase susceptibility to skeletal Class III malocclusion diseases (we suggest assessing and performing GWAS using more than five thousand human samples with Class III malocclusion and ten thousand control samples). Currently, extensive molecular research is being carried out on the regulatory RNAs, including gene expression, DNA methylation, small and microRNA, and long non-coding RNA profiles, about numerous illnesses. To our knowledge, there has been little research on the role of these chemicals in skeletal Class III malocclusion. In this paper, we argue that these regulatory RNAs will be crucial to studying this illness, leading to a greater understanding of the disease’s molecular underpinnings. The pipeline flowchart for producing system genetic datasets of cellular, molecular, and clinical trait data combined to analyze various correlations between malocclusion and Class III phenotypes is embodied in Figure 2. The flow diagram of the selection process of the Class III malocclusion in man, along with its identification, screening, and exclusion methods, is represented in Figure 3.

## 5. Conclusions

Skeletal Class III malocclusion is a complex condition with diverse phenotypic features that can adversely affect physical, social, and psychological well-being. The diagnosis of skeletal Class III malocclusion is crucial in determining the most suitable treatment approach based on the patient’s growth potential. Currently, there are three procedures for correcting skeletal Class III malocclusion, including growth modification, orthodontic camouflage therapy, and surgical orthodontics. Mandibular prognathism is a complex trait that can result from both genetic and environmental factors. Identifying the genetic factors and understanding their mechanisms with Class III malocclusion development would aid in the diagnosis, prediction, and treatment of skeletal variations. Overall, further research is needed to advance the understanding of the genetics and treatment options for skeletal Class III malocclusion.

It is believed that the complex nature of the genetic factors involved in skeletal Class III malocclusion cannot be unraveled using only approaches designed to identify the main effects of individual genotypes/alleles in humans but requires a multi-dimensional research approach. Therefore, it is necessary that future studies focus on generating a new Class III malocclusion model that provides a good model that includes a large, well-characterized, and diverse subject population to identify the genetic causes and study the factors that lead to the diversity and heterogeneity of this condition in humans more comprehensively. This model should incorporate a wide range of genomic and molecular techniques and advanced phenotyping approaches.

We propose that including a large subject population consisting of a well-characterized, multi-ethnic sample, including individuals with European, Middle Eastern, Hispanic/Latino, admixed African, and Asian ancestry, will increase the power of the analysis and the opportunity to comprehensively identify genetic causes for skeletal Class III malocclusion development in man.

Finally, here, we are extending an open invitation to national and international orthodontic clinicians and orthodontic surgery physicians to join in this effort by providing DNA samples from skeletal Class III malocclusion patients, following the approved Helsinki ethical protocol so that we can challenge these phenomena through a joint effort.

In summary, our recommendation is for a coordinated and multidisciplinary research effort to address the genetic complexities of skeletal Class III malocclusion. This will require the participation of diverse populations, cutting-edge technology, and collaborative partnerships to provide new insights into the pathogenesis of this condition and to develop personalized treatments for affected individuals.

## Figures and Tables

**Table 2:** The top 20 enriched skeletal Class III malocclusion-associated pathways.

Pathway Identifier	Pathway Name	Submitted Entities Found
R-HSA-5663202	Diseases of signal transduction by growth factor receptors and second messengers	*HDAC4*; *JAG1*; *WNT3A*; *PSEN2*; *FOXO3*; *DUSP6*; *FGF3*; *NCOR2*; *ERLEC1*; *FGF20*; *EP300*; *FGF23*; *FGFR2*; *FGFR1*
R-HSA-1226099	Signaling by FGFR in disease	*FGF20*; *FGF23*; *FGFR2*; *FGF3*; *FGFR1*
R-HSA-1839126	FGFR2 mutant receptor activation	*FGF20*; *FGF23*; *FGFR2*; *FGF3*
R-HSA-2428928	IRS-related events triggered by IGF1R	*FGF20*; *IGF1*; *FGF23*; *FGFR2*; *FGF3*; *FGFR1*
R-HSA-2428924	IGF1R signaling cascade	*FGF20*; *IGF1*; *FGF23*; *FGFR2*; *FGF3*; *FGFR1*
R-HSA-2404192	Signaling by Type 1 Insulin-like Growth Factor 1 Receptor (IGF1R)	*FGF20*; *IGF1*; *FGF23*; *FGFR2*; *FGF3*; *FGFR1*
R-HSA-5655253	Signaling by FGFR2 in disease	*FGF20*; *FGF23*; *FGFR2*; *FGF3*
R-HSA-109704	PI3K Cascade	*FGF20*; *FGF23*; *FGFR2*; *FGF3*; *FGFR1*
R-HSA-112399	IRS-mediated signaling	*FGF20*; *FGF23*; *FGFR2*; *FGF3*; *FGFR1*
R-HSA-5654221	Phospholipase C-mediated cascade; FGFR2	*FGF20*; *FGF23*; *FGFR2*; *FGF3*
R-HSA-190241	FGFR2 ligand binding and activation	*FGF20*; *FGF23*; *FGFR2*; *FGF3*
R-HSA-74751	Insulin receptor signaling cascade	*FGF20*; *FGF23*; *FGFR2*; *FGF3*; *FGFR1*
R-HSA-5654695	PI-3K cascade:FGFR2	*FGF20*; *FGF23*; *FGFR2*; *FGF3*
R-HSA-5654699	SHC-mediated cascade:FGFR2	*FGF20*; *FGF23*; *FGFR2*; *FGF3*
R-HSA-5654700	FRS-mediated FGFR2 signaling	*FGF20*; *FGF23*; *FGFR2*; *FGF3*
R-HSA-157118	Signaling by NOTCH	*NCOR2*; *HDAC4*; *NOTCH3*; *JAG1*; *NOTCH4*; *PSEN2*; *NUMB*; *EP300*
R-HSA-2219528	PI3K/AKT signaling in cancer	*FGF20*; *FOXO3*; *FGF23*; *FGFR2*; *FGF3*; *FGFR1*
R-HSA-2219530	Constitutive Signaling by aberrant PI3K in cancer	*FGF20*; *FGF23*; *FGFR2*; *FGF3*; *FGFR1*
R-HSA-74752	Signaling by insulin receptor	*FGF20*; *FGF23*; *FGFR2*; *FGF3*; *FGFR1*
R-HSA-5654727	Negative regulation of FGFR2 signaling	*FGF20*; *FGF23*; *FGFR2*; *FGF3*

## Data Availability

Not applicable.
